# Sublingual Immunotherapy Decreased the Serum Levels of Interleukin-36*γ* in Allergic Rhinitis

**DOI:** 10.1155/2024/9692031

**Published:** 2024-11-06

**Authors:** Xiaowei Qin, Chunrui Wang, Jueqi Li, Xiaopeng Zhang, Tianhong Zhang

**Affiliations:** Department of Otolaryngology, The First Affiliated Hospital of Harbin Medical University, Harbin, China

**Keywords:** allergic rhinitis, IL-36*γ*, sublingual immunotherapy

## Abstract

**Background:** Allergy immunotherapy (AIT), a treatment approach for allergic rhinitis (AR), is recognized for its potential to modify the disease course beyond mere symptom relief. Interleukin-36*γ* (IL-36*γ*), a key player in immune responses, has been implicated in promoting eosinophilic inflammation in AR by activating eosinophils. We aimed to investigate the effect of IL-36*γ* on group II lymphoid cell (ILC2) in AR patients who underwent sublingual immunotherapy (SLIT).

**Methods:** Twenty-four AR patients were enrolled and administered with SLIT. Serum proteins of IL-36*γ*, interleukin-5 (IL-5), and interleukin-13 (IL-13) during SLIT were quantitatively assessed using enzyme-linked immunosorbent assay (ELISA). The proportion of ILC2 was determined by flow cytometry. Sorted ILC2s were stimulated by IL-36*γ* and ILC2 cell differentiation, and type II cytokines expression were examined.

**Results:** SLIT treatment decreased the serum protein levels of IL-36*γ*, IL-5, IL-13, and the proportion of ILC2 significantly. IL-36*γ* suppressed the proliferation of ILC2 by inhibiting the levels of ILC2 transcription factor. IL-36*γ* also inhibited IL-5 and IL-13 expression from ILC2.

**Conclusion:** The changes of IL-36*γ* during SLIT were related to the inhibited function of ILC2, implying that IL-36*γ* may be used as a new biomarker for monitoring the efficacy of SLIT in AR.

## 1. Introduction

Allergic rhinitis (AR), a prevalent condition characterized by eosinophilic inflammation of the nasal mucosa, is accompanied by the infiltration of T-help 2 (Th2) cells and excessive mucus production, significantly impacting patients' quality of life [1].

Allergy immunotherapy (AIT) is believed to alter the disease's progression, reduce reliance on medication, and maintain therapeutic benefits even after cessation of treatment [2]. AIT included either subcutaneously (SCIT) or sublingually (SLIT), both of which have been proven effective and safe in numerous studies [3–5]. Previous studies found that AIT regulates the immune balance by shifting the immune response from a Th2-dominated inflammation to T-help 1 (Th1) inflammation [6]. Meanwhile, the eosinophilic inflammation was alleviated after SLIT accompanied by elevated Immunoglobulin G4 (IgG4) levels [6]. Interleukin-36*γ* (IL-36*γ*) on group II innate lymphoid cells (ILC2s), known for their role in allergy through the secretion of Th2 cytokines, have also been found to be inhibited by AIT in AR patients [7–9].

IL-36*γ* has been proven to play an important role in bridging innate and adaptive immunity [10]. For example, IL-36*γ* is upregulated and involved in neutrophil infiltration in psoriasis by activating the endothelium and promoting lymphocyte recruitment [11, 12]. In chronic rhinosinusitis, IL-36*γ* production and activation may enhance neutrophilic inflammation. Our previous research has demonstrated that IL-36*γ* is also involved in eosinophilic inflammation in AR, activating eosinophils [13].

Given these insights, we aim to delve into the dynamics and effects of IL-36*γ* during SLIT in AR patients.

## 2. Methods

### 2.1. Patients

A retrospective study was conducted at our hospital, spanning from August 2020 to August 2022. A total of 24 adult participants with a confirmed diagnosis of AR were recruited for this study. The diagnosis of AR was established in accordance with the guidelines outlined in the AR and its Impact on Asthma (ARIA) document. The diagnostic criteria included typical allergic manifestations, such as rhinorrhea, nasal pruritus, sneezing, nasal obstruction, a positive skin-prick test (SPT) result, and/or the presence of serum-specific immunoglobulin E (IgE) antibodies reactive to *Dermatophagoïdes farina*, either singly or in conjunction with *Dermatophagoïdes pteronyssinus*. Participants were excluded if they had a history of asthma, nasal polyposis, immunodeficiency, severe systemic illnesses, or sensitivities to allergens other than *D. farina* or *D. pteronyssinus*.

For SLIT, *D. farinae* drops (manufactured by Zhejiang Wolwo, China) were administered. The dosage regimen involved a gradual escalation of concentrations, ranging from 1 to 333 μg/mL, over a 3-week period, adhering to the recommended schedule of 1, 2, 3, 4, 6, 8, and 10 drops of formulations numbered 1 through 3, respectively, each week for 3 consecutive weeks. During the maintenance phase, commencing from the 4th week onward, a constant dose of 333 μg/mL (administered as three drops) was given until the completion of the therapeutic course.

### 2.2. Efficacy Evaluation

Allergic symptoms were recorded and graded as follows: 0, no symptoms; 1, slight symptoms; 2, moderate symptoms; 3, severe symptoms. The medication usage was scored as follows: 1, oral antihistamine or nasal antihistamine; 2, intranasal corticosteroid. The patients who obtained a 30% reduction in symptom and medication scores than baseline scores were enrolled in the response group.

### 2.3. Flow Cytometry and Sorting for ILC2

Peripheral blood mononuclear cells (PBMCs) were isolated through density-gradient centrifugation using Lymphoprep (Fresenius Kabi Norge AS, Oslo, Norway) from heparinized leucocyte-enriched buffy coats. The obtained PBMCs were subsequently cultured at a concentration of 2 × 10^6^ cells/mL in 24-well plates, supplemented with Roswell Park Memorial Institute (RPMI) 1640 medium containing 5% human albumin (AB) serum, 5 mmol/L glutamine, and a solution of penicillin and streptomycin (all reagents were sourced from Invitrogen, with the exception of the serum which was obtained from Sigma–Aldrich). PBMCs were stained by lineage markers (cluster of differentiation 3 [CD3], CD14, CD16, CD19, CD20, CD56, Fc*ε*R1*α*) and antibodies to chemoattractant receptor homologous molecule (CRTH2) and CD127 antibody. The ILC2 cells (Lin^−^CRTH2^+^CD127^+^ cells) were purified using FACSAria (BD Biosciences).

To examine the impact of IL-36*γ* on these purified ILC2 cells, various concentrations of IL-36*γ* (ranging from 10 to 100 ng/mL) were introduced into the culture system. To quantify the proliferative response of the ILC2 cells to these stimuli, we employed a tritium-labeled thymidine incorporation assay, which allowed us to measure the extent of cell division and, thus, the proliferative capacity of the ILC2 population under different conditions.

### 2.4. Real-Time Polymerase Chain Reaction (PCR) Analysis

RNA was extracted from ILC2 with RNeasy kit (Venlo). The expression of GATA binding protein 3 (GATA3) (5′-GCGGGCTCTATCACAAAATGA-3′, 5′-GCTCTCCTGGCTGCAGACAGC-3′) and receptor-related orphan receptor *α* (ROR*α*) (5′-AAGGAGCCAGAAGGGATGAAC-3′, 5′-GGAACA ACAGACGCCAGTAAG-3′) were detected using an ABI 7300 System (Applied Biosystems). The messenger RNA (mRNA) levels of the detected genes were normalized to the housekeeping gene glyceraldehyde-3-phosphate dehydrogenase (GAPDH). This normalization process was achieved through the comparative *Δ*Ct method.

### 2.5. Enzyme-Linked Immunosorbent Assay (ELISA)

The expression of IL-36*γ* protein was detected by ELISA kits (R&D Systems, DY2320-05). The detection limit of IL-36*γ* was 18.8 pg/mL.

### 2.6. Statistical Analysis

All data are presented as the medians and interquartile ranges. The nonparametric Mann–Whitney *U* test was done for comparison between the two groups, except additional note. One-way analysis of variance (ANOVA) followed by Dunnett's test was performed for comparison among more than two groups. Spearman rank correlation analysis was used for correlation analysis. A *P* < 0.05 was defined as statistically significant.

## 3. Results

### 3.1. SLIT Decreases IL-36*γ* Expression and Proportion of ILC2

The demographic information of patients is summarized in Table 1. SLIT administration resulted in a significant reduction in both symptom severity and medication usage scores (Table 2). After 2 years' SLIT, the serum protein levels of IL-36*γ*, interleukin-5 (IL-5), interleukin-13 (IL-13), and the proportion of ILC2 were inhibited significantly (Table 3). The protein levels of IL-36*γ*, IL-5, IL-13, and the proportion of ILC2 in the responsive group were significantly lower than in the unresponsive group (Table 4). The IL-36*γ* expression was significantly correlated with the ILC2 ratio.

### 3.2. IL-36*γ* Regulated ILC2 Proliferation and Function

**Figure 1:**
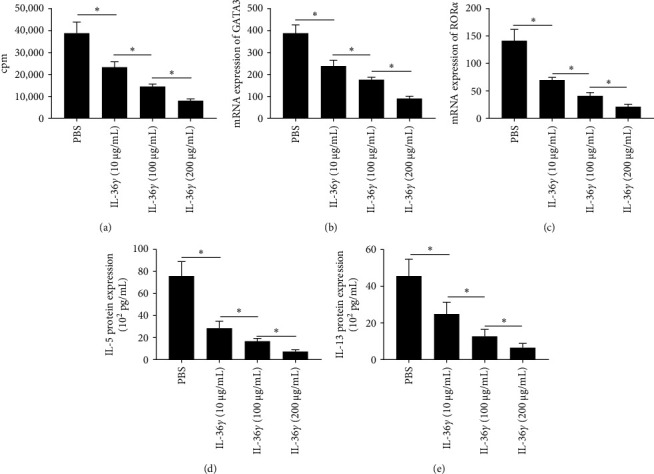
IL-36*γ* regulated ILC2 proliferation and function: (A) IL-36*γ* decreased ILC2 proliferation determined by tritiated thymidine incorporation. (B, C) The mRNA levels of GATA3 and ROR*α* by ILC2. (D, E) The protein expression of IL-5 and IL-13 by ILC2. *⁣*^*∗*^*P* < 0.05. cpm, counts per minute; GATA3, GATA binding protein 3; IL-5, interleukin-5; IL-13, interleukin-13; IL-36*γ*, interleukin-36*γ*; ILC2, IL-36*γ* on group II lymphoid cell; mRNA, messenger RNA; PBS, phosphate-buffered saline; ROR*α*, receptor-related orphan receptor *α*.

In vitro experiments revealed that IL-36*γ* exerts a suppressive effect on ILC2 proliferation by downregulating the expression of key transcription factors, GATA3, and retinoid acid ROR*α*. Furthermore, the levels of IL-5 and IL-13 secreted by IL-36*γ*-stimulated ILC2s were significantly reduced compared to unstimulated controls, as illustrated in Figure 1.

## 4. Discussion

AIT has emerged as an effective therapeutic approach for managing AR and asthma [14, 15]. Several studies have revealed a transition from a Th2-dominated response to a Th1-prevalent pattern subsequent to SLIT treatment, and this process involves complex immune regulatory mechanisms. For example, Ciprandi's study suggested that a decrease in the expression of human leukocyte antigen (HLA)-G, HLA-A, HLA-B, and HLA-C molecules appears to be intricately linked to one of the postulated mechanisms underlying the efficacy of SLIT, particularly its ability to redirect the immune system's response from a Th2 cytokine secretion pattern towards a Th1-oriented one [16].

Recently, studies suggested that AIT can also reduce the proportion of ILC2 in house dust mite (HDM) or grass pollen-sensitized AR patients [8, 9]. Moreover, AIT also reduced IL-13 expression by ILC2 in AR patients during the pollen season, as well as seasonal symptoms [8]. Mitthamsiri's study found a significant reduction of circulating ILC2 and elevation of ILC1 after AIT, which is similar to the proportion as seen in controls [17]. However, one study failed to demonstrate a change in ILC2 proportions in grass pollen-allergic AR patients following AIT [18].

In our study, we also found that SLIT treatment decreased ILC2 proportion in HDM-sensitized AR patients. Moreover, the serum levels of IL-36*γ*, IL-5, IL-13, and ILC2 proportion in the responsive group were significantly lower compared with the unresponsive group, suggesting that alleviation of symptoms during SLIT may be related to decreased proportion of ILC2.

IL-36*γ*, a member of the IL-1 cytokine family, has been well-established as a key mediator in various inflammatory reactions [10]. Its receptor, IL-36R, is expressed by a diverse array of immune and non-immune cells, including dendritic cells, T cells [19–23], endothelial cells [24], keratinocytes [25, 26], intestinal epithelial cells, and fibroblasts. Elevated IL-36*γ* expression has been implicated in the pathogenesis of inflammatory bowel disease (IBD) [27, 28] and asthma models, where it is induced in bronchial epithelial cells upon exposure to viral infections, smoke, or allergens [29, 30].

To substantiate this relationship, we conducted in vitro experiments demonstrating that IL-36*γ* directly inhibits ILC2 proliferation and modulates the expression of key transcription factors. Furthermore, IL-36*γ* suppressed the production of type II cytokines by ILC2s, highlighting its direct effect on ILC2 function.

Interestingly, our previous study suggested that the upregulation of IL-36*γ* in nasal epithelial cells is facilitated by IL-17, IL-25, and IL-33 [13]. Similarly, in normal bronchial epithelial cells, IL-36*γ* expression can be enhanced in response to double-stranded RNA, IL-1*β*, tumor necrosis factor-*α* (TNF-*α*), and IL-17 stimuli [31]. It is universally acknowledged that IL-17, IL-25, and IL-33 can promote the proliferation and function of ILC2, but on the other hand, they induce epithelial cells to produce IL-36*γ*, which in turn inhibits the proliferation and function of ILC2 cells, thus forming a negative feedback loop.

The disparate responsiveness to these stimuli may stem from the varied expression patterns of IL-36R within the nasal cavity. Intriguingly, allergen exposure, specifically to *D. pteronyssinus* group 1 (Der p1) from HDM, significantly upregulates IL-36R expression on eosinophils isolated from AR patients' peripheral blood and neutrophils purified from healthy individuals [13]. These findings underscore the potential significance of IL-36*γ* as a crucial pathway in modulating AR inflammation, particularly through its regulatory effects on eosinophils, which are key effector cells in allergic inflammatory responses.

## 5. Conclusion

Our study is the first to demonstrate a relationship between changes in IL-36*γ* levels during SLIT and the inhibition of ILC2 function. Our findings propose IL-36*γ* as a novel biomarker for SLIT efficacy, offering a potential predictive tool for personalized treatment strategies in AR management.

## Figures and Tables

**Table 1:** Demographic characteristics of AR patients.

Characteristics	SLIT group
Number	24
Sex (male:female)	13:11
Age (months)	28.9 ± 11.6
Baseline symptoms (TNSS)	9.9 ± 2.8

Abbreviations: AR, allergic rhinitis; SLIT, sublingual immunotherapy; TNSS, total nasal symptom score.

**Table 2:** Decreased symptom and medication score before and after 2 years' SLIT.

Scores	Baseline	Two years' SLIT
Symptom score	7.1 ± 2.1	2.2 ± 0.8*⁣*^*∗*^
Medication score	6.0 ± 1.9	3.4 ± 1.0*⁣*^*∗*^
Total score	12.8 ± 3.6	5.6 ± 2.2*⁣*^*∗*^

Abbreviation: SLIT, sublingual immunotherapy.

*⁣* ^*∗*^Compared with baseline, *P* < 0.05.

**Table 3:** Decreased cytokine levels and ILC2 proportion after 2 years' SLIT.

Characteristics	Baseline	Two years' SLIT
IL-36*γ* (pg/mL)	956.3 ± 315.2	413.2 ± 159.8*⁣*^*∗*^
IL-5 (pg/mL)	22.5 ± 8.9	11.3 ± 5.4*⁣*^*∗*^
IL-13 (pg/mL)	295.1 ± 78.5	75.3 ± 21.6*⁣*^*∗*^
Proportion of ILC2 (%)	0.13 ± 0.05	0.03 ± 0.01*⁣*^*∗*^

Abbreviations: IL-5, interleukin-5; IL-13, interleukin-13; IL-36γ, interleukin-36γ; ILC2, IL-36γ on group II lymphoid cell; SLIT, sublingual immunotherapy.

*⁣* ^*∗*^Compared with baseline, *P* < 0.05.

**Table 4:** Comparison of scores and cytokine levels between responsive and unresponsive groups.

Characteristics	Responsive group	Unresponsive group
Symptom score	1.8 ± 0.6*⁣*^*∗*^	5.9 ± 2.4
Medication score	2.8 ± 1.1*⁣*^*∗*^	5.6 ± 1.8
Total score	4.7 ± 1.7*⁣*^*∗*^	10.5 ± 3.3
IL-36*γ* (pg/mL)	377.5 ± 159.1*⁣*^*∗*^	838.1 ± 347.8
IL-5 (pg/mL)	9.6 ± 4.8*⁣*^*∗*^	24.1 ± 9.3
IL-13 (pg/mL)	68.2 ± 23.9*⁣*^*∗*^	273.4 ± 81.6
Proportion of ILC2 (%)	0.02 ± 0.01*⁣*^*∗*^	0.14 ± 0.04

Abbreviations: IL-5, interleukin-5; IL-13, interleukin-13; IL-36γ, interleukin-36γ; ILC2, IL-36γ on group II lymphoid cell.

*⁣* ^*∗*^Compared with baseline, *P* < 0.05.

## Data Availability

The data that support the findings of this study are available from the corresponding author upon reasonable request.
